# Qualitative analysis of the impact of top surgery on physical activity in transmasculine and non-binary adolescents and young adults: a pilot study

**DOI:** 10.1007/s00431-026-06897-4

**Published:** 2026-05-12

**Authors:** Kirtana Sandepudi, Brenda Haddad, Cole Roblee, Rebecca Arteaga, Annaliese Krausse, Jaina Gaudette, Christopher Eyo, Paige Hackenberger, Diane Chen, Lauren Beach, Sumanas W. Jordan

**Affiliations:** 1 Division of Plastic & Reconstructive Surgery, Northwestern University Feinberg School of Medicine, Chicago, IL USA; 2 Rosalind Franklin University of Medical Sciences, North Chicago, IL USA; 3 Potocsnak Family Division of Adolescent and Young Adult Medicine, Pritzker Department of Psychiatry and Behavioral Health, Ann & Robert H. Lurie Children’s Hospital of Chicago, Chicago, IL USA; 4 Departments of Psychiatry & Behavioral Sciences, and Pediatrics, Northwestern University Feinberg School of Medicine, Chicago, IL USA; 5 Department of Medical Social Sciences, Northwestern University Feinberg School of Medicine, Chicago, IL USA

**Keywords:** Chest dysphoria, Top surgery, Physical activity, Transgender and non-binary, Adolescents, Young adults

## Abstract

Physical activity (PA) is crucial in adolescents and young adults (AYA) for the promotion of long-term cardiovascular health. In transgender and non-binary (TGNB) AYA who were assigned female at birth (AFAB), chest dysphoria may lead to avoidance of sports and exercise. Thus, it is important to understand the impact of gender-affirming top surgery on PA in TGNB AFAB AYA. In this pilot study, we conducted a qualitative analysis of the associations among chest dysphoria, top surgery, and the relationship with PA. Two focus groups, each consisting of five TGNB AFAB patients who were 13–25 years old at the time of top surgery, were conducted to elicit perspectives on factors impacting PA, including top surgery. Focus group transcripts were analyzed using grounded theory coupled with situational analysis. Identified themes were grouped into barriers, facilitators, and complicating factors of PA. Most barriers, including structural accessibility, passing, binding/clothing restrictions, and physical safety, were prominent prior to top surgery. Several facilitators to PA arose after top surgery, including top surgery improving PA, social acceptance, and PA to augment the post-op chest. Complicating factors included mental health, which impacted PA both before and after surgery, and the post-op healing process, which impacted PA for up to 6 months post-surgery.

*Conclusion*: In TGNB AFAB adolescents and young adults, chest dysphoria poses several barriers to PA, many of which are resolved by top surgery. These findings suggest that alleviation of chest dysphoria via gender-affirming care has implications for the long-term cardiovascular health of TGNB patients.

**Table:** 

** What is Known: **
• *Chest dysphoria can be a barrier to physical activity in transgender and non-binary (TGNB) adolescents and young adults (AYA)*.
• *Physical activity in adolescence can shape longer term cardiovascular health*.
** What is New: **
• *Gender-affirming top surgery resolves many barriers to physical activity in TGNB AYA*.
• *Our findings suggest top surgery can have implications for long-term cardiovascular health*.

## Introduction

Chest dysphoria, an emotional and physical discomfort with one’s chest anatomy, is a common and distressing experience among transgender and non-binary (TGNB) people assigned female at birth (AFAB) [1]. Among youth, chest dysphoria becomes particularly salient during puberty as breasts develop [1]. Chest dysphoria has been linked to higher self-reported anxiety and depression [2] which can subsequently negatively affect physical health. Specifically, chest dysphoria has been associated with lower quality of life in transmasculine adolescents and young adults (AYA), including avoidance of sports and exercise due to difficulty of chest concealment [3]. Furthermore, in an analysis comparing TGNB and cisgender high school-aged youth, TGNB youth had lower rates of preventative checkups, highlighting potential disparities in healthcare utilization [4]. One approach to managing chest dysphoria is chest binding (wearing a constrictive garment to flatten the chest). Additionally, exogenous testosterone hormone therapy is commonly sought by TGNB AFAB individuals to address general gender dysphoria. However, these therapies can have limitations in their efficacy to address chest dysphoria. Prior research has revealed that chest binding induces pain, skin irritation and difficulty breathing, and that hormone therapy does not meaningfully address chest dysphoria due to chest anatomy largely remaining the same [1, 3]. Top surgery, in the form of gender-affirming mastectomy or breast reduction, in TGNB AFAB AYA has been shown to reduce chest dysphoria [5] and improve body image, gender congruence, and quality of life, leading to improved mental health and physical well-being [5, 6].

Promoting physical activity (PA) during adolescence plays a crucial role in shaping long-term health. A review by Hallal et al*.* highlights that PA in adolescence positively impacts adult PA, bone health and self-esteem [7]. PA is also known to prevent cardiovascular disease, which has become increasingly prevalent among young adults, along with obesity and diabetes [8, 9]. Although there is a paucity of literature assessing motivators and barriers to PA in TGNB AYA, extensive research has been conducted in cisgender AYA. An analysis of the national Youth Risk Behavior Surveillance Survey (YRBS) showed that female students had lower levels of PA than male students among high school-aged youth [10]. Among female students, Black and Hispanic students had lower levels of PA compared to non-Hispanic White students [10]. A study investigating PA of girls and boys aged 12–16 at Australian secondary schools found that girls were less likely to participate in organized sports and were more likely to experience teasing related to their bodies [11]. Despite this lower overall participation, the study highlighted that drive for thinness, body shame, and appearance anxiety were linked to increased PA in girls. Boys were often motivated by a drive for increased muscularity [11].

In the TGNB population, literature about factors affecting PA is limited. In adults, a study by Jones et al. found that TGNB people undergoing hormone therapy experienced multiple barriers to PA, including body dissatisfaction, fear of not being accepted, and discomfort in typical athletic clothing [12]. Facilitators to PA included seeing changes in their body and staying healthy for future gender-affirming surgery [12]. Another recent analysis of YRBS data in American high school-aged youth revealed that people who identified as transgender and reported their sex assigned at birth as female were significantly less likely to participate in PA compared to female non-transgender youth [13]. Adjusting for bullying did not significantly alter results, suggesting that factors other than bullying, which are not captured by YRBS, may impact PA in transgender high school-aged youth [13].

There remains a gap in the literature regarding the impact of chest dysphoria and top surgery on PA among TGNB AFAB AYA. Therefore, the purpose of this pilot study was to better understand how top surgery influences PA in TGNB AFAB AYA. Our team led two focus groups and conducted a grounded theory analysis to explore participants’ perspectives on associations between chest dysphoria, top surgery, and the relationship with PA.

## Methods

This study was conducted in accordance with the Declaration of Helsinki and under the approval of Northwestern’s Institutional Review Board (STU#00210403). Inclusion criteria included the following: (1) AFAB people who identify as TGNB, (2) were age 13–25 years at the time of gender-affirming top surgery, (3) underwent surgery in the last 5 years, and (4) speak English. Participants were recruited using flyers and an online consent form, after which study team members reached out to coordinate focus groups. Parental consent was required for participants under 18 years old.

Two semi-structured focus groups of five participants each were conducted on Zoom, following a pre-established interview guide developed with input from TGNB study team members under the guidance of the senior author, a gender-affirming surgeon (SWJ). The aim of the focus groups was to elicit perspectives on the individual level of PA (“What physical activity do you currently participate in, if any?”), factors affecting engagement in physical activity (“What factors do you believe play a role in your engagement with physical activity?”), difficulties with participating in PA (“Have you ever encountered barriers to participation in physical activity because of how society perceived your body?”), and the impact of chest dysphoria and top surgery on PA (“How does chest dysphoria affect your engagement in physical activity? Has this changed after top surgery?”). Focus groups lasted approximately 60 min. Participants were compensated with a $50 online gift card.

Focus groups were transcribed by carefully cleaning auto-generated Zoom transcripts. Cleaned transcripts were uploaded into Dedoose [14]. Open coding and preliminary codebook preparation were conducted by 4 coders. A grounded theory approach [15] was employed to identify themes due to the lack of existing frameworks describing physical activity in TGNB AYA. Once an initial codebook was agreed upon by the study team, it was entered into Dedoose. Inter-rater reliability testing was conducted in Dedoose by having two coders assign codes to the same set of excerpts inclusive of the full list of identified themes. Once the kappa reported in Dedoose was 0.8, the two coders independently coded the two transcripts. Only codes that applied to more than one participant were retained in the final codebook. Axial coding was guided by Clarke’s situational analysis [16] as seen in prior literature analyzing PA in TGNB adults [12]. A grounded theory was developed, and the time-based relationship between undergoing top surgery, gender affirmation, and PA was mapped. Four TGNB people were involved in coding and thematic analysis. Finally, our authors come from varying disciplines (including two surgeons, one pediatric psychologist, and one medical social sciences researcher with expertise in qualitative methods), ensuring interpretation of data was not skewed toward one set of preconceptions.

## Results

### Participant demographics

Of the ten participants, five identified as transgender men and four identified as non-binary. One participant identified as “other,” specifying “man with trans experience.” The average age at the time of surgery was 19.7 years old (range 13–24, SD 3.5), and the average age at the time of participation was 21.7 years old (range 14–26, SD 4.0). There were six white participants, one Asian participant, one mixed white and Asian, one who answered “other,” and one who did not answer. Two participants identified as Hispanic or Latinx. There was no significant difference in any demographics between the two focus groups (Table 1).

**Table 1:** Demographics of focus group participants

	Focus group 1	Focus group 2	*p*-value
*Age*			
At time of surgery (SD)	18.6 (3.8)	20.8 (3.1)	0.18
At time of participation (SD)	20.2 (4.1)	23.2 (3.6)	0.13
*Gender identity*			
Trans men (%)	3 (60%)	2 (40%)	0.36
Non-binary or other (%)	2 (40%)	3 (60%)	
*Ethnicity*			
Hispanic/Latinx	1 (20%)	1 (20%)	–
Not Hispanic/Latinx	4 (80%)	4 (80%)	
*Race*			
White	3	3	–
Asian	0	1	
Mixed	1	0	
Other	0	1	
Did not answer	1	0	

### Identified themes

**Fig. 1:**
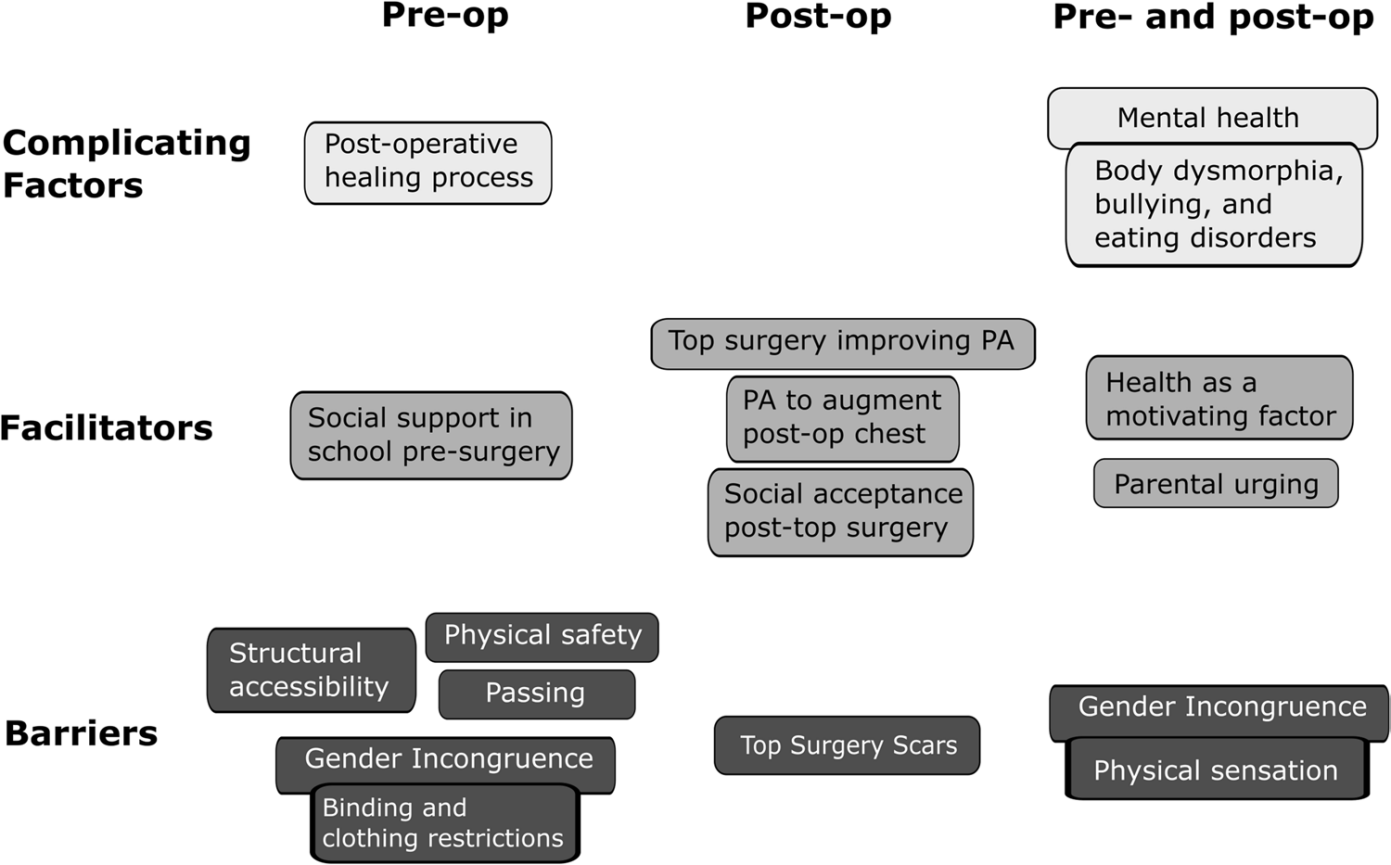
Summary of axial coding developed using situational analysis, revealing the time-based relationship between top surgery and physical activity. Shading is used to distinguish themes that represent barriers (dark), facilitators (medium), and complicating factors (light). See Tables 2, 3, and 4 for details about each theme

Analysis of focus group transcripts revealed several themes, which were classified under three axial codes—barriers, facilitators, and complicating factors of PA (Tables 2, 3, and 4). Complicating factors were defined as a distinct axial code because they could lead to either increased or decreased PA and may have complex interplay with other aspects of health. Theme reporting below is organized according to the development of the team’s grounded theory that was generated following Clarke’s positional mapping process (Fig. 1). After analysis of both focus groups, there were no distinct themes identified in focus group 2 compared to the first group, suggesting saturation had been reached.

**Table 2:** Part 1 of codebook derived from focus group transcripts using ground theory approach, focusing on barriers to physical activity, with exemplar quotes and associated participant demographics (gender identity and age range in years). Participant IDs are in Px.y format, where “x” indicates focus group number and “y” indicates participant’s assigned number within their focus group. *TM* transgender man, *NB* non-binary. Race was not included because it would be identifying

Parent code	Child code	Grandchild code	Description	Exemplar quote(s)
Physical activity: barriers	Gender Incongruence as a barrier to PA	Chest dysphoria may lead to barriers or distress during the pre-operative timeline, impeding an individual’s ability to conduct physical activities		Q1: “But it was just kind of a pain, because I felt like I always had to be like hyper vigilant and hyper aware of, like everything that’s going on at any time and then also seeing like my classmates like chests because they had their shirts off and then feeling almost inferior because I didn’t have that same body type or even like body structure just sort of like really, it was not a fun time.” P1.5, TM, 13–17 y
		Binding and Clothing Restrictions	Gender incongruence may lead to binding or clothing choices that interfere with the ability to engage in physical activity	Q2: “I know in the summers I get really hot a lot and wearing of the binder, and wanting to go swimming—I love swimming. So I felt like I had to actually put like I put a binder, and then 2 swim shirts on, and then that would make me very uncomfortable. And I feel like I just never did well with that.” P2.5, TM, 13–17 y
		Physical Sensation	Gender incongruence may lead to discomfort with the physical sensation of the chest, which often is a barrier to physical activity	Q3: “I would say that I experienced the same thing before top surgery, that, like, I never wanted to run or jog or anything because of, like, the feeling of my chest. And even, like, for a little bit after I had top surgery when I was healing, I would still feel the same sensation, like, especially in the car, like, going over bumps and that caused me, like, a lot of dysphoria. And I would say that was like the biggest hindrance for me doing physical activity before surgery.” P1.4, NB/other, 22–25 y
	Passing	The possibility of being perceived as a gender that is not aligned with one’s identity may deter people from participating in physical activity		Q4: “I mean, not having a chest like that in the beginning? Would’ve changed everything! Of course it would have, you know? If I had not had second puberty, if I had gone on blockers and like T, when I was a teenager that would have been completely a different story. But yeah, in like masculine workout spaces for sure, I feel like a fraud for sure.” P2.2, TM, 22–25 y Q5: “And it’s just like less stressful when thinking about wanting to exercise or something, because it’s like I don’t have to wear like a tight sports bra, it’s like my chest doesn’t move or anything. So it’s just way easier to do physical activity and not have to worry about like, how my being perceived? Or how is this making me feel dysphoria? It just feels natural and less anxious, like less anxiety-inducing.” P2.4, NB/other, 18–21 y
	Structural accessibility	Lack of structural accessibility, including but not limited to availability of inclusive locker room spaces, poses a barrier to physical activity		Q6: “So I like just would completely avoid going into locker rooms or going to the bathroom when I was at the gym. And yeah, just feeling a lot more like cautious, or like anxious about what other people are perceiving me as. As opposed to now, when I go to the gym like I’ll go to the men’s locker room. It’s gross, but like I feel comfortable being in there than I did before. And yeah, I just feel like a lot less anxiety.” P2.1, NB/other, 22–25 y Q7: “…there was only like a very few select people at the gym who knew I was trans […] But it always felt like if we were doing like handstands or whatever and my shirt would fall down like I was like that would be the end of the world, for me, like […] a lot of the members are very conservative and like that doesn’t necessarily mean they’re…like trans phobic or whatever, but there’s definitely like this stereotype, um, so it makes me a little uncomfortable” P1.5, transmasculine, 13–17 y
	Top Surgery Scars	Scars from top surgery may hinder physical activity, either due to physical sensation and pain in early healing process or due to visibility of scars in masculine workout environments		Q8: “I, for lack of better term, sometimes it feels as if, I don’t know how to say this, but sometimes it feels like the scar is going to rip open and then that will definitely scare me into stopping what I’m doing. Or even if I’m doing push up sometimes and I’m in that planking position for too long, I’ll feel it in the actual scar, the scarring, and then I’ll stop, so that’s a hindrance for me at times.” P1.1, NB/other, 18–21 y
	Physical Safety	Fears and threats related to physical safety pose a barrier to physical activity		Q9: “I quite literally had on like a big giant T shirt like the biggest pair of like shorts that I had I would pack I would make sure that I like… 100% passed or like if I like walked into a big man or something like I wouldn’t get beaten up to death or something um and.” P1.2, TM, 18–21 y
		Binding problems	Binding, which is commonly done during physical activity, can potentially leading to physical harm. Thus, TGNB youth are detered from engaging in PA	Q10: “I would say like before, it was like hard to exercise just because I had a bigger chest, so I would wear my binder 24/7. So like even like walking for a while like it got to the point where it hurt to breathe a lot.” P2.4, NB/other, 18–21 y

**Table 3:** Part 2 of codebook derived from focus group transcripts using ground theory approach, focusing on facilitators to physical activity, with exemplar quotes and associated participant demographics (gender identity and age range in years). Participant IDs are in Px.y format, where “x” indicates focus group number and “y” indicates participant’s assigned number within their focus group. *TM* transgender man, *NB* non-binary. Race was not included because it would be identifying

Parent code	Child code	Grandchild code	Description	Exemplar quote(s)
Physical activity: facilitators	Health as a motivating factor for PA	Individuals may feel motivated to engage in physical activity in order to maintain their overall health		Q11: “So, yeah, me wanting to be active also is because I want to remain healthy and like stay in good shape and keep my, my health good and that kind of thing because, you know, it catches up to you […] so that’s why I want to stay active.” P1.1, NB/other, 18–21 y Q12: “All of the guys my family are pretty big and my dad literally just had, just got diagnosed with diabetes so it’s a really big thing for me to try and keep my health as good as I can” P1.2, TM, 18–21 y
	Parental urging	Parents may encourage their children to engage in physical activity		Q13: “I would say, like, it’s kind of funny because when I’m in, like, a really bad mood I won’t want to go exercise, but, of course, like my parents are like “no go exercise anyway,” so I end up doing it, and I feel a lot better.” P1.5, TM, 13–17 y
	Top Surgery Improving PA Post-Op	Top surgery may increase frequency and quality of physical activity		Q14: “After I’ve gotten top surgery I’m way more active, like running a lot more than I ever had in my life, like going to a gym, which I was kind of not doing before, and kind of afraid of doing before. So yeah, having top surgery definitely increased my physical activity. “ P2.1, NB/other, 22–25 y Q15: “but I’m like six months post op now… And I feel like I’m even stronger now than I was before, probably, probably in part due to confidence getting more comfortable at the gym and being willing to push boundaries, a little bit more” P1.5, TM, 13–17 y
	PA to augment Post-Op Chest	Physical activity might be used to augment the appearance of the post-op chest		Q16: “It’s on my left side that’s a little more saggy than my right because my left side was a little bigger. So recently what I’ve been doing is I’ve been doing, like chest workouts at home and doing a lot of like pre workout stuff before even like going to work like within the beginning of the week, and that sort of kind of helped.” P1.2, TM, 18–21 y
	Social Acceptance Post-Top Surgery	Social acceptance in various settings may increase following top surgery		Q17: “I’m just a regular trans guy, but I’m a little fruity. You know, like my gender expression has gotten more, more femme, maybe, after being on T, after being on top surgery, because, like I can be. I’m just a guy who does what he wants. I’m allowed to now, versus like, if I did that before anything, I think people would definitely misgender me.” P2.2, TM, 22–25 y
	Social Support in School Pre-Top Surgery	Peers	Social support from peers reduces barriers to physical activity participation in schools	Q18: “Um so with me, one of the big problems, especially through high school with having a bigger chest, is that when I would have to change in the locker rooms, of course, I had, you know, my friends that have known me for years that like were right next to me, so I felt very, very comfortable with them, but they would also like create like a circle for me so like when I would change like nobody would like see.” P1.2, TM, 18–21 y
		Teachers	Social support from teachers reduces barriers to physical activity participation in schools	Q19: “And they would always be like okay for two teams we’re going to have shirts and skins and I’d always be on the skins and I’d be like, oh no. Luckily like my gym teacher would come over and be like, no, no, no, no we’re not doing this and then all the kids would be confused as to why we couldn’t take our shirts off” P1.5, TM, 13–17 y

**Table 4:** Part 3 of codebook derived from focus group transcripts using ground theory approach, focusing on complicating factors of physical activity, with exemplar quotes and associated participant demographics (gender identity and age range in years). Participant IDs are in Px.y format, where “x” indicates focus group number and “y” indicates participant’s assigned number within their focus group. TM = transgender man; NB = non-binary. Race was not included because it would be identifying

Parent code	Child code	Grandchild code	Description	Exemplar quote(s)
Complicating factors	Mental Health	Negative mental health experiences or manifestations may impede an individual's engagement with physicial activity, regardless of surgical status		Q20: “I didn’t really do much exercise when I was younger. But I wish I did know about top surgery before others, because like I didn’t realize how unhappy it made me until, well, until after.” P2.5, TM, 13–17 y
		Body dysmorphia	Individuals may worry about perceived flaws in their appearance that are unrelated to gender dysphoria	Q21: “Um besides that, I would say, like, it’s not the healthiest motivation, but I’m, like, really self conscious about my appearance and I never got diagnosed with an eating disorder, but I’ve had, like, body dysmorphia and have restricted intake due to that um so it’s not always the healthiest motivation but it’s motivation.” P1.5, TM, 13–17 y
		Bullying	Bullying often causes mental distress, intefering with one’s ability to participate in activities, particularly in school	Q22: “I’m not sure if anybody here is like gone through the whole high school experience or not, you know doing like senior year and all of that, um the bullying for me at least socially got very, very bad to the point where I had to graduate high school early, because if I didn’t, there was no way I was going to survive my senior year.” P1.2, TM, 18–21 y
		Eating disorders	Eating disorders may complicate one’s motivation or ability to exercise	Q23: “I’ve also struggled with an eating disorder so that’s also played a role in my physical activity and, like, why I do physical activity.” P1.3, TM, 13–17 y
	Post-op healing process and PA	The post-operative healing period, which usually lasts a few months after top surgery, can make it difficult to engage in physical activity		Q24: “So it was like 3 months before I felt confident, to start actually lifting more than like 10 pounds, or to actually try to like, run around or go out and do fun things.” P2.4, NB/other, 18–21 y Q25: “I was probably back to more or less normal after 6 weeks. […] Because for me at least, it was a very gradual, from being like I’m scared I’m gonna hurt myself, to like this just feels weird, to eventually at some point your body just feels normal again. And I mean normal for me was definitely several months, if not maybe a year or more, out. But that didn’t prevent me from doing the activities I normally would, it just felt weird.” P2.3, non-binary/other, 18–21 y

#### Barriers to physical activity

##### Pre-top surgery

Prior to top surgery, participants cited gender incongruence, specifically in relation to binding and clothing restrictions, as a barrier to PA. Many described feeling uncomfortable engaging in PA unless wearing a binder or baggy clothing that could conceal the shape of their body. This feeling explicitly hindered participation in sports that require wearing fitted or revealing clothing, such as swimming. Additionally, although wearing baggy clothing was not entirely prohibitive, it did make intense PA such as running uncomfortable (Table 2, 1–3).

I know in the summers I get really hot a lot and wearing of the binder, and wanting to go swimming - I love swimming. So I felt like I had to actually put like I put a binder, and then 2 swim shirts on, and then that would make me very uncomfortable. And I feel like I just never did well with that. P2.5, transmasculine, 13–17 y

Participants also noted that anxiety around passing (a term which may refer to being perceived as a cisgender person of one’s affirmed gender) [17, 18] prior to top surgery often deterred them from engaging in PA in masculine workout spaces (Table 2, Q4–5). For many TGNB individuals, passing has high importance in spaces where they might feel unsafe if perceived as transgender [17].

I mean, not having a chest like that in the beginning? Would've changed everything! Of course it would have, you know? If I had not had second puberty, if I had gone on blockers and like T, when I was a teenager that would have been completely a different story. But yeah, in like masculine workout spaces for sure, I feel like a fraud for sure. P2.2, transmasculine, 22–25 y

Similarly, structural accessibility, including availability of appropriate locker room spaces, lack of trans-friendly gym spaces, and binary gendered sports in school was also a concern among many participants (Table 2, Q6–7). Participants relayed a need to lean on social support from peers and teachers to circumvent the challenges posed by the lack of inclusiveness in locker room spaces and organized sports. This role of social support is detailed further in the facilitators section below.

So I like just would completely avoid going into locker rooms or going to the bathroom when I was at the gym. And yeah, just feeling a lot more like cautious, or like anxious about what other people are perceiving me as. As opposed to now, when I go to the gym like I’ll go to the men's locker room. It's gross, but like I feel comfortable being in there than I did before. P2.1, non-binary or other, 22–25 y

Finally, participants highlighted the interplay between fear for physical safety and PA prior to top surgery. They reported fear of being harmed by others in masculine spaces and unintentional physical harm due to wearing a binder for prolonged periods of time (Table 2, Q9-10).

I would say like before, it was like hard to exercise just because I had a bigger chest, so I would wear my binder 24/7. So like even like walking for a while like it got to the point where it hurt to breathe a lot. P2.4, non-binary or other, 18–21 y

##### Post-top surgery

For up to 6 months after top surgery, participants felt that their scars hindered PA. This occurred for a variety of reasons, including pain or physical discomfort in the region of the scar and anxiety that the scar may open from repeated activity if not fully healed (Table 2, Q8). Some participants expressed discomfort around others seeing their scars, while others felt confident having their scars visible in public spaces.

##### Both before and after surgery

Participants reported that dysphoria due to the physical sensation of their chest prevented them from engaging in PA. This experience was common prior to top surgery, when participants avoided activities that could accentuate the physical sensation of having female breasts, such as running, jumping jacks, or wrestling. After surgery, some participants noted residual dysphoria due to physical sensation, limited to the post-operative healing period (Table 2, 3).

I would say [...] before top surgery, that, like, I never wanted to run or jog or anything because of, like, the feeling of my chest. And even, like, for a little bit after I had top surgery when I was healing, I would still feel the same sensation, like, especially in the car, like, going over bumps and that caused me, like, a lot of dysphoria. And I would say that was like the biggest hindrance for me doing physical activity before surgery. P1.4, non-binary or other, 22–25 y

#### Facilitators of physical activity

##### Pre-top surgery

Before undergoing top surgery, participants noted that social support was an important facilitator of PA participation, particularly in school. Both staff and students were able to make informal accommodations that allowed TGNB students to safely participate in gym class while avoiding uncomfortable or unsafe situations while changing clothes or participating in organized sports. For example, one participant recounted that his friends would form a circle around him while changing in the boys’ locker room to prevent others from seeing his chest (Table 3, Q18). Another stated gym teachers and staff would interject when his classmates suggested the typical “shirts vs. skins” team system in boys’ sports (Table 3, Q19).

When I would have to change in the locker rooms, of course, I had, you know, my friends that have known me for years that like were right next to me, so I felt very, very comfortable with them, but they would also like create like a circle for me so like when I would change like nobody would like see. P1.2, transmasculine, 18–21 y

##### Post-top surgery

Following top surgery, PA was facilitated by several factors, including increased social acceptance and comfort during exercise. Social acceptance allowed participants to feel comfortable using spaces such as locker rooms or pools (Table 3, Q17). An increase in the frequency of activity following surgery was often noted (Table 3, Q14–15). Participants also sought out PA such as weightlifting to augment their post-operative chest and achieve their desired appearance (Table 3, Q16).

After I’ve gotten top surgery I’m way more active, like running a lot more than I ever had in my life, like going to a gym, which I was kind of not doing before, and kind of afraid of doing before. So yeah, having top surgery definitely increased my physical activity. P2.1, non-binary or other, 22–25 y

##### Both before and after surgery

Both before and after surgery, participants were motivated to engage in PA through factors unrelated to surgical status. These included the desire to maintain a healthy lifestyle, and urging from parents (Table 3, Q11–13).

So, yeah, me wanting to be active also is because I want to remain healthy and like stay in good shape and keep my, my health good and that kind of thing because, you know, it catches up to you [...] so that’s why I want to stay active. P1.1, non-binary/other, 18–21 y

#### Complicating factors of physical activity

##### Post top surgery

After top surgery, participants noted post-op healing as a complicating factor to PA. While many felt motivated to engage in PA after surgery, participants often reported the healing process delaying their return to activities (Table 4, Q24–25).

So it was like 3 months before I felt confident, to start actually lifting more than like 10 pounds, or to actually try to like, run around or go out and do fun things. P2.4, NB/other, 18–21 y

##### Both before and after surgery

Participants reported mental health as a complicating factor to PA both before and after top surgery (Table 4, Q20). Specifically, participants cited body dysmorphia, or self-consciousness around their physical appearance unrelated to gender dysphoria, as something that might motivate or prevent them from exercising (Table 4, Q21). Bullying and eating disorders were also reported as mental stressors which complicated their ability to engage in PA (Table 4, Q22–23).

Um besides that, I would say, like, it’s not the healthiest motivation, but I’m, like, really self-conscious about my appearance and I never got diagnosed with an eating disorder, but I’ve had, like, body dysmorphia and have restricted intake due to that um so it’s not always the healthiest motivation but it’s motivation. P1.5, transmasculine, 13–17 y

## Discussion

In this study, we qualitatively assessed the impacts of top surgery on PA among TGNB AFAB AYA who had received gender-affirming top surgery between the ages of 13 and 25 years. Using semi-structured focus group interviews and a grounded theory approach coupled with situational analysis, we found that in TGNB AFAB adolescents and young adults, chest dysphoria was associated with multi-level barriers to PA, many of which were alleviated by gender-affirming top surgery via reduction in chest dysphoria (Fig. 1).

Our study uncovered several barriers to PA in this population, including gender incongruence, structural accessibility, and concerns about passing and physical safety. Many of these barriers were resolved after top surgery. Previous quantitative surveys administered to both transgender adults and young patients undergoing hormone therapy similarly found themes of body dissatisfaction, discomfort in athletic clothing, and fear of not being accepted as barriers to PA [12, 19]. A recent qualitative study looking broadly at experiences related to top surgery and chest dysphoria uncovered themes related to lack of accessibility in public restrooms, avoidance of sports, and high levels of dysphoria prior to top surgery. They also found that top surgery led to improved overall quality of life [3]. Our study builds on these findings and illustrates the unique impact of these barriers on PA in AYA.

While some participants felt confident showing their scars, others avoided PA due to their scars. Reasons for this included discomfort around the visibility of scars or anxiety that movement could cause scars to pull open, even six months into recovery. Previous literature echoes these mixed sentiments toward appearance and physical sensation of scars among patients who have received top surgery [3, 20]. From a clinical perspective, several factors including surgical approach, initial breast size, and skin laxity can impact the appearance of top surgery scars [21, 22]. Additionally, patients are advised to avoid PA for only about 4–6 weeks after surgery, when incisions are fully healed [23]. Thus, it is crucial for clinicians to incorporate patient education about the healing timeline and consider scar concealment in their pre-operative counseling where appropriate.

Our study also highlighted important facilitators to PA in TGNB AYA. Before top surgery, one important facilitator that participants noted was social support in school. Previous studies have called attention to the importance of social support in school, particularly from key adults such as teachers [24]. We found that this is also key in the context of gym class, where supportive peers and teachers were able to circumvent situations in which appropriate infrastructure does not exist for TGNB youth, such as changing in locker rooms or wrestling in gym class. This social support in the school environment can also be seen as an example of community-based resilience as described by Meyer’s sex and gender minority stress/resilience model [25].

No previous studies have investigated the impact of top surgery on PA in transgender and nonbinary AYA populations. Studies on TGNB adults have demonstrated that transmasculine adults have a high degree of motivation for PA to improve gender congruence and to improve health for potential surgery in the future [12]. Gender-affirming hormone therapy was also found to increase PA levels [26]. Our study builds on these existing findings to not only show that chest augmentation and a desire to improve health act as facilitators for PA in TGNB youth, but also that top surgery improves PA in a similar manner to other gender-affirming care.

Several of these facilitators have also been demonstrated among cisgender adolescents. In our study, TGNB AYA sought out PA to augment their post-operative chest. Previous studies have shown that cisgender boys pursue activities such as weightlifting out of a drive for “muscularity” or a traditionally masculine appearance [11]. Drivers to PA including overall health, parental encouragement, and PE teacher support have also been well-demonstrated in cis populations [11]. Our study illustrates that these motivators are also present in TGNB AYA, both before and after top surgery.

Our findings underscore the complex interplay between mental health and PA, both before and after top surgery, with emphasis on body dysmorphia and eating disorders in participants who were 13–17 years old at the time of surgery. Although seemingly independent of top surgery, mental health and eating disorders are important factors to consider in pre- and post-operative counseling. Eating disorders have a peak age of onset between ages 14 and 19 in the general population [27], and gender dysphoria has been observed to increase risk, especially in transmasculine adolescents [28]. Importantly, prior literature has demonstrated that eating disorders, especially anorexia nervosa, can lead to a problematic increase in use of PA. This pattern of behavior is heavily influenced by other co-morbid mental health conditions including depression and anxiety [29]. Our findings highlight the importance of multidisciplinary gender-affirming care practices. In these settings, surgeons, psychologists, primary care providers, and social workers can collaborate to provide comprehensive pre-operative care that addresses mental health and eating disorders.

It is important to consider these results in the context of the larger clinical picture. Increasing numbers of adolescents and young adults are seeking gender-affirming top surgery [30], making it more important and urgent to understand the ways in which top surgery impacts the health of this population. Additionally, adolescence and early adulthood are crucial ages during which intervention can have immense long-term health impact. Adolescent PA is a well-established predictor of adult PA [7, 31]. The association between levels of PA in adulthood and risk of cardiovascular disease is also well-demonstrated [32]. Therefore, factors that impact PA in adolescence and early adulthood are an important consideration when optimizing long-term health.

## Limitations

This study had several limitations that must be acknowledged. First, with this being a pilot study and a minoritized population, our sample size was limited to ten participants. Additionally, all our participants were from a single geographic area and interviewed within a one-year time period. Thus, while this study elucidated several important themes related to the impact of top surgery, the transferability of our results to the whole TGNB AFAB AYA population is limited. We intend to continue exploring the topic of PA in other TGNB AYA cohorts.

The racial and ethnic diversity of our sample population was also limited. Our group intends to expand on our findings in future work to explore the intersectionality of gender identity, race, ethnicity, and other social determinants of health in shaping PA.

## Implications and contribution

In TGNB AFAB adolescents and young adults, chest dysphoria poses several barriers to physical activity, many of which are resolved by gender-affirming top surgery. These findings suggest that alleviation of chest dysphoria via gender-affirming care has implications for the long-term cardiovascular health of TGNB patients.

## Data Availability

Data is provided within the manuscript, but full transcripts are not included to protect privacy of participants. Any questions about data presented in the study, or requests for additional data, may be directed to the corresponding author.

## Abbreviations

AFAB: Assigned female at birth
AYA: Adolescents and young adults
PA: Physical activity
TGNB: Transgender and non-binary
YRBS: Youth Risk Behavior Surveillance Survey
